# Sentinel Lymph Node Dissection—Novelty, Trend, or a Paradigm Shift in Surgical Decision-Making for Early Cervical Cancer?

**DOI:** 10.3390/medicina61091660

**Published:** 2025-09-12

**Authors:** Angel Yordanov, Eva Tsoneva, Ihsan Hasan, Stoyan Kostov

**Affiliations:** 1 Department of Gynecologic Oncology, Medical University Pleven, 5800 Pleven, Bulgaria; 2 Department of Reproductive Medicine, Specialized Hospital for Active Treatment of Obstetrics and Gynaecology “Dr Shterev”, 1330 Sofia, Bulgaria; dretsoneva@gmail.com; 3 Department of Obstetrics and Gynecology, University Hospital “Sofiamed”, 1000 Sofia, Bulgaria; ihsan_hasanov@abv.bg; 4 Research Institute, Medical University Pleven, 5800 Pleven, Bulgaria; drstoqn.kostov@gmail.com; 5 Department of Gynecology, Hospital “Saint Anna”, Medical University—“Prof. Dr. Paraskev Stoyanov”, 9002 Varna, Bulgaria

**Keywords:** cervical cancer, sentinel lymph node, sensitivity, cost effectiveness, recurrences, survival rate

## Abstract

Cervical cancer remains the fourth most common malignancy among women worldwide, with over 600,000 new cases and approximately 350,000 deaths in 2022. Lymph node (LN) status is a critical prognostic factor, and in 2018, the International Federation of Gynecology and Obstetrics (FIGO) revised its staging system to include regional LN metastases, underscoring the importance of accurate nodal assessment. Sentinel lymph node biopsy (SLNB) has emerged as a minimally invasive alternative to systematic pelvic lymphadenectomy in early-stage disease, aiming to shorten operative time, reduce healthcare costs, and minimize treatment-related morbidity. This review synthesizes current evidence on SLNB in early-stage cervical cancer, including its diagnostic accuracy, optimal techniques, cost-effectiveness, and remaining clinical challenges. Data from prospective trials and meta-analyses demonstrate that SLNB provides high detection rates, especially with bilateral mapping and the use of advanced tracers such as indocyanine green. Ultrastaging further improves the detection of micrometastases and isolated tumor cells, refining adjuvant therapy decisions. Compared to full lymphadenectomy, SLNB significantly decreases intraoperative blood loss, operative time, and postoperative complications—most notably, lymphedema—while maintaining equivalent disease-free and overall survival. International guidelines now endorse SLNB for appropriately selected patients with early-stage cervical cancer (tumor size < 4 cm, negative preoperative imaging). However, variations persist between European and U.S. recommendations regarding its role as a standalone procedure. Future research must address protocol standardization, the prognostic relevance of low-volume metastases, and factors influencing mapping success. Overall, SLNB represents a paradigm shift toward more individualized, evidence-based surgical management of early-stage cervical cancer.

## 1. Introduction

Cervical cancer remains the fourth most common malignancy among women globally, with approximately 660,000 new cases and 350,000 deaths recorded in 2022 [1]. The incidence of the disease varies significantly across regions—while rates are declining in high-income countries, they are on the rise in low- and middle-income settings [2]. As cervical cancer predominantly affects younger women, it has a considerable social impact; it is estimated that around 20% of children who lose their mothers to cancer are affected by this specific diagnosis [3].

The status of lymph nodes (LNs) is recognized as an independent prognostic factor. In 2018, the International Federation of Gynecology and Obstetrics (FIGO) updated its staging system to include the presence of regional lymph node metastases [4]. Determining nodal involvement is essential when planning treatment. Patients with metastatic lymph nodes are managed with chemoradiotherapy, whereas surgery is generally reserved for early-stage disease (FIGO stages IA1 to IIA1, with tumor size up to 4 cm) without lymph node spread. In some specific cases, surgery may be required for LN-negative patients with T1b3 and T2a2 tumors, but the surgery has to be performed only in highly specialized centers [4].

Standard surgical management for early-stage cervical cancer includes simple/radical hysterectomy and pelvic lymphadenectomy (for staging purposes, paraaortic LN dissection can be performed) [4,5]. If LN metastases are found on final histology, patients are referred for adjuvant chemoradiotherapy [4,5]. However, data suggest that only 15–20% of patients in early stages have nodal metastases, indicating that the majority undergo pelvic lymphadenectomy without therapeutic benefit [6].

This has led to growing interest in sentinel lymph node biopsy (SLNB) as a way to reduce overtreatment. SLNB has the potential to shorten operative time, lower healthcare costs, and reduce the risk of complications, such as vascular or nerve injury, lymphoceles, lymphedema, and lymphatic leakage. It also allows for ultrastaging and may help identify sentinel nodes in atypical anatomical locations.

The sentinel lymph node (SLN) is the first LN to receive drainage from the primary tumor and is considered a predictor of the status of the remaining nodal basin. This principle is already well established in other malignancies, such as breast cancer and melanoma [7]. If the sentinel node is free of metastases, a full pelvic lymphadenectomy might be safely avoided while still obtaining reliable staging information. This approach has become standard in other cancer types [8,9]

The method of SLN detection depends on the tracer used. With dyes, the first colored node is identified, while with radioactive tracers such as technetium-99 m, the “hot” node is detected using a gamma probe. Despite numerous studies supporting the high sensitivity of SLNB in cervical cancer, clinical practice remains inconsistent, especially regarding subsequent management after SLN detection [10,11,12,13,14,15].

European guidelines—issued by the European Society of Gynecological Oncology (ESGO), the European Society for Radiotherapy and Oncology (ESTRO), and the European Society of Pathology (ESP)—recommend intraoperative frozen section analysis of the SLN. This allows the surgical team to proceed with pelvic lymphadenectomy if the SLN is negative or to discontinue surgery and refer the patient for definitive chemoradiotherapy if metastasis is confirmed [16]. It is not recommended to use SLNB as a standalone procedure.

In contrast, the U.S.-based National Comprehensive Cancer Network (NCCN) guidelines support the use of SLNB as a standalone procedure in selected early-stage cases (IA1 with lymphovascular space invasion, IA2, IB1, IB2, and IIA1). They note that the most reliable results are seen in tumors less than 2 cm in size [17].

The first reports on the use of SLNB in cervical cancer emerged in the early 1990s, using techniques such as blue dye, technetium-99 m, or a combination of both for node localization [18,19,20,21,22,23,24].

While the accumulated evidence supports the safety and diagnostic value of SLNB, several important clinical questions remain unanswered. These include the following:

1. In which clinical scenarios of early-stage cervical cancer should SLNB be performed?
2. What is the preferred surgical approach for SLNB—minimally invasive or open surgery?
3. Which tracer is most appropriate for intraoperative identification of the SLN, and what is the optimal technique for its administration?
4. Can SLNB be replaced by preoperative imaging modalities for LN assessment?
5. What is the detection rate of SLNB for identifying sentinel lymph nodes in early-stage cervical cancer?
6. What are the most common anatomical locations of SLNs in patients with early-stage cervical cancer?
7. What is the recommended intraoperative management strategy when no SLN is identified?
8. How should excised SLNs be examined—via intraoperative frozen section or paraffin-embedded tissue sections? Are there discrepancies between these two diagnostic methods?
9. What is the clinical relevance of ultrastaging in SLN assessment, particularly regarding micrometastases (MMs) and isolated tumor cells (ITCs)?
10. Does performing SLNB intraoperatively influence oncological outcomes and overall survival?
11. What is the cost-effectiveness ratio of SLNB, and how should procedural costs be calculated, especially in relation to histopathological processing and evaluation?
12. Is there an association between SLNB and the incidence of early or late postoperative complications?
13. Are there specific risk factors—such as high BMI, prior conization, or tumor size—that may serve as contraindications to intraoperative SLNB?

## 2. Methodology

We identified a narrative review type of article as the most appropriate for focusing on a broad range of particular questions from different perspectives.

The review and the consensus process were performed between January 2025 and March 2025. A detailed and comprehensive literature search of articles (studies written in English, French, and German) regarding SLNB in cervical cancer was performed. We conducted a computer-based, extensive review of the following databases: Google Scholar, Cochrane Library, SciELO, and publishers’ databases (Elsevier/ScienceDirect, Wiley, Wolters Klouwer/Lippincott, Taylor & Francis, Springer, Sage, Hindawi, Termedia, and Via Medica). We used the following keywords and Medical Subject Headings (Mesh) terms: “cervical cancer”, “sentinel lymph node”, “sentinel lymph node biopsy”, “micrometastasis”, “frozen section”, “sensitivity”, “lymph node assessment”, “adverse effects”, “treatment”, “cost effectiveness”, “lymph node dissection”, “risk factors”, “postoperative complications”, “recurrences”, and “survival rate”. References from recent review papers were scanned to identify other related articles. Mainly articles from the 21 st century were included. Only a few old articles (dedicated mainly to the history of SLNB) were incorporated. The final selection of references was performed after full-text reading. We included all types of articles (original research, systematic reviews, meta-analyses, narrative reviews, and case reports). The majority of the publications had a retrospective nature due to the rarity of the disease. Some studies were repeated because they were considered in different contexts, but we tried not to repeat information in the different subsections of this manuscript.

### Strength of Evidence

To assist readers in interpreting the strength of the studies cited, we qualitatively categorized them within the text. Randomized controlled trials, such as SENTICOL II and FILM, represent the highest level of evidence. Prospective cohort studies, like SENTIREC, SENTIX, and SCCAN, provide robust support for clinical practice. Retrospective studies and single-institution case series offer valuable observations, although they are limited by design. Finally, recommendations from expert bodies, such as ESGO and NCCN, reflect current consensus and standard-of-care guidelines. No institutional review board or ethics committee approval was required due to the review nature of this article.

## 3. Discussion

### 3.1. In Which Cases of Early-Stage Cervical Cancer Should SLNB Be Performed?

In the early years of implementing SLNB as an intraoperative technique for the identification of SLNs, the procedure was primarily applied in patients with early-stage cervical cancer—specifically FIGO stages IA, IB1, and IB2, which corresponded to tumors up to 4 cm in size without involvement of adjacent structures, according to the FIGO 1998 classification [18,19,23]. Some authors, however, extended its application to more advanced cases, such as FIGO stage IIA [20,24,25,26]. Even at that time, a notable inverse correlation was observed between tumor size and SLNB sensitivity—the method demonstrated higher detection rates in tumors smaller than 4 cm [20].

In 2008, the AGO Study Group reported findings from their SLNB experience in patients ranging from FIGO stages IA1 to IVB (per the 1998 classification) [11]. Their data revealed significantly higher detection rates in tumors ≤ 2 cm compared to those > 2 cm (94.0% vs. 83.6%). The tracers used for SLN mapping included technetium-99 m (99 mTc), patent blue dye, or a combination of both.

These findings were not entirely corroborated by Salvo et al., who employed 99 mTc, patent blue dye, or a combination of either with indocyanine green (ICG) or ICG alone [27]. Similar outcomes were reported in the SENTIREC clinical trial, which also included patients with tumors > 4 cm, where ICG was used for intraoperative SLN visualization and detection [28].

Currently, SLNB is recommended as a standalone procedure by NCCN for patients with tumors < 4 cm in size [29]. It is important to note that the majority of clinical studies have been conducted in cohorts with tumors ≤ 4 cm [13,30,31].

At present, SLNB is considered appropriate for all patients eligible for surgical treatment—namely, those in FIGO stages IA to IB2 and IIA1, with tumor size < 4 cm according to the 2018 FIGO classification. Larger tumors are generally not candidates for surgical management, and thus, SLNB is not the procedure of choice in such cases, despite isolated reports demonstrating its feasibility in locally advanced cervical cancer [32,33,34]. Nonetheless, several studies have documented a significantly reduced SLN detection rate in these advanced stages [35,36,37]. As such, while SLNB may be technically feasible in tumors up to 4 cm, optimal performance is observed in tumors smaller than 2 cm [38,39,40,41,42].

### 3.2. What Is the Optimal Surgical Approach for Performing SLNB?

SLNB can be performed either via open surgery or through minimally invasive approaches, such as laparoscopy or robot-assisted surgery [43]. From the earliest applications of SLNB, many authors have utilized laparoscopy as the preferred method for SLN identification [18,21].

However, the publication of the LACC (Laparoscopic Approach to Cervical Cancer) trial significantly shifted the perspective on surgical access. The trial demonstrated a clear oncological advantage of open surgery over minimally invasive techniques. Specifically, the 4.5-year disease-free survival rate was 96.5% for open surgery vs. 86.0% for minimally invasive surgery—a difference of −10.6 percentage points (95% confidence interval [CI], −16.4 to −4.7). The 3-year disease-free survival was 97.1% vs. 91.2%, with an HR for recurrence or death from cervical cancer of 3.74 (95% CI, 1.63–8.58). Overall 3-year survival also favored the open approach—99.0% compared to 93.8%, with an HR for death from any cause of 6.00 (95% CI, 1.77–20.30) [44].

In light of these findings, minimally invasive techniques for SLNB should currently be limited to clinical trial settings. To date, there are no robust comparative studies directly evaluating SLN detection rates according to surgical access. Nevertheless, a meta-analysis that included 49 studies with 2476 patients found no statistically significant differences in detection success between open, laparoscopic, and robot-assisted techniques. Specifically, the sensitivity and detection rates reported were as follows:

- Laparotomy: sensitivity of 0.86 (95% CI, 0.80–0.90); detection rate of 0.87 (95% CI, 0.83–0.91);
- Laparoscopy: sensitivity of 0.90 (95% CI, 0.86–0.94); detection rate of 0.93 (95% CI, 0.90–0.96);
- Robot-assisted surgery: sensitivity of 0.84 (95% CI, 0.72–0.92); detection of rate 0.92 (95% CI, 0.88–0.95) [45].

These findings suggest that the overall performance of SLNB is not significantly influenced by the choice of surgical approach. Rather, differences in outcomes reported across studies are more likely attributable to other factors, such as the type of tracer used, injection technique, and institutional experience. Therefore, it may be concluded that the surgical route itself is not a critical determinant of SLN detection success.

### 3.3. What Tracer Should Be Used for Intraoperative Sentinel Lymph Node Detection and How Should It Be Administered?

Initial reports on SLNB described the use of various dyes—including patent blue, isosulfan blue, or lymphazurin—administered alone or in combination with radiotracers such as 99 mTc. Over time, ICG has increasingly emerged as a preferred tracer, often used as a standalone agent.

A 2014 meta-analysis including 49 studies evaluated the sensitivity and detection rates associated with different tracer techniques [45]:

- Combined techniques (dye + 99 mTc): sensitivity of 0.88 (95% CI, 0.84–0.91); detection rate of 0.97 (95% CI, 0.96–0.98);
- Technetium-99 m alone: sensitivity of 0.87 (95% CI, 0.78–0.93); detection of rate 0.90 (95% CI, 0.87–0.93);
- Blue dye alone: sensitivity of 0.87 (95% CI, 0.79–0.93); detection rate of 0.87 (95% CI, 0.84–0.90).

According to current guidelines by ESGO, SLN detection should be performed either using a combination of blue dye and 99 mTc or using ICG alone [46]. The use of blue dye as a sole tracer is discouraged due to its lower detection efficacy [46]. While ICG has been shown to outperform isosulfan blue dye [47], it does not demonstrate superiority over the combined dye–radioisotope technique [48]. Nevertheless, two meta-analyses report that ICG offers the highest detection rates when compared to blue dye, 99 mTc, or their combination [49,50].

**Figure 1:**
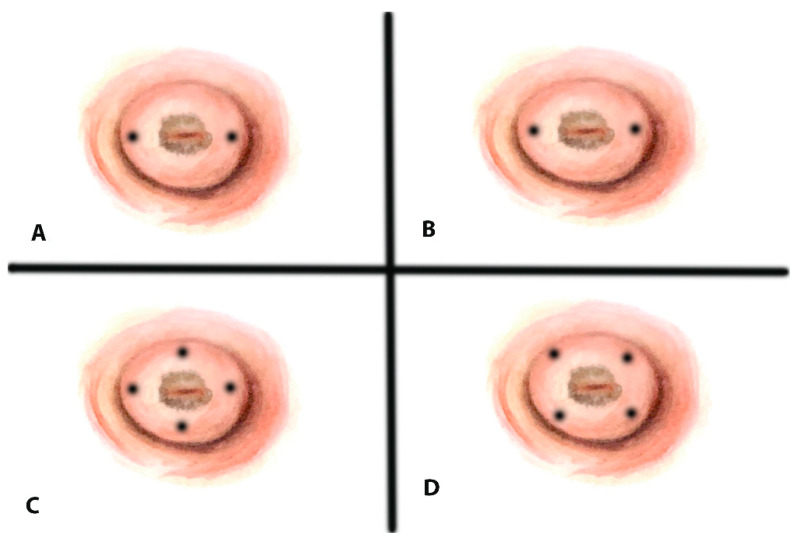
Injection sites for the tracer agent: (**A**) two superficial points; (**B**) two deep points; (**C**) four cardinal points; (**D**) four diagonal points.

Tracer injection is typically performed into the cervix, either around the tumor periphery or at the site of a previous conization scar. Several injection protocols are used, most commonly involving either two-point (3 and 9 o’clock) or four-point injections (see Figure 1) [43].

When the two-point injection technique is selected, the tracer is typically administered both superficially and deeply (at depths of 5 mm and 20 mm, respectively) [51]. Injection of blue dye or ICG is performed immediately prior to the surgical procedure to prevent the tracer from migrating beyond the SLN and staining downstream nodes, which may be mistakenly identified as additional sentinel nodes.

Radiotracer 99 mTc is injected superficially according to two main protocols: the extended protocol involves administering 120 MBq on the day before surgery, with a maximum interval of 15 h before the operation; the short protocol involves injecting 60 MBq on the morning of surgery. In both protocols, preoperative lymphoscintigraphy is feasible—performed 3 to 5 h after injection for the extended protocol and 1 to 3 h after injection for the short protocol [43].

Based on the available evidence, the use of ICG appears to be the most appropriate option, while the injection technique should be adapted at the discretion of the operating surgeon.

### 3.4. Can Preoperative Imaging Techniques Replace Intraoperative SLNB?

With advancements in imaging modalities, the ability to detect metastatic LNs has significantly improved. While magnetic resonance imaging (MRI) remains the optimal choice for evaluating the primary tumor [52,53], positron emission tomography (PET) has emerged as the leading modality for LN assessment [54]. PET/CT demonstrates superior sensitivity and specificity (73% and 98%, respectively) compared to MRI (56% and 93%) and computed tomography (CT) (58% and 92%) [55]. However, multiple studies report inferior negative predictive values (74–88% vs. 97–100%), sensitivities (0–68% vs. 75–96.3%), and specificities (84–98% vs. 94–100%) for PET/CT when compared to SLN biopsy [28,56]. These findings indicate that, at present, SLN biopsy cannot be reliably substituted by any imaging modality.

### 3.5. What Is the Detection Rate of SLNB for Identifying SLNs?

The evaluation of SLNB performance should distinguish between overall SLN detection (i.e., at least unilateral) and bilateral detection, as well as the tracer used. Key metrics also include sensitivity, specificity, false negative rate (FNR), and negative predictive value (NPV) (Table 1).

This table presents comparative data on detection rates, sensitivity, false negative rates, and negative predictive value (NPV) across major prospective studies and meta-analyses.

- The SENTICOL study (n = 139, tumors ≤ 4 cm) using a combined tracer approach (blue dye + 99 mTc) reported a detection rate of 97.8% overall (95% CI, 93.8% to 99.6%) and 76.5% bilaterally, with a sensitivity of 92.0%, an NPV of 98.2% (95% CI, 74.0% to 99.0%), and an FNR of 1.4% [13].
- Kim et al. used ICG in 103 women with stages IA1 (LVSI+) to IIA, finding SLNs in 100% of the cases and bilaterally in 85.44%. The reported sensitivity was 76.92% (95% CI, 57.95–88.97%), the sensitivity was 100% (95% CI, 95.00–100%), the FNR was 23.08%, and the NPV was 92.41% (95% CI, 84.40–96.47%) [58]. For tumors < 2 cm without radiographic lymphadenopathy, the sensitivity and specificity reached 100% (95% CI 20.65–100% and 95% CI, 94.42–100%) [57].
- Dostálek et al. (n = 350) used blue dye + radiocolloid and found an overall detection rate of 93%, with bilateral detection in 80%. The sensitivity was 93–96%, with an FNR of 1.6–0% depending on the tumor size subgroup (<2 cm, 2–4 cm, >4 cm) [58].
- The SENTIREC trial used ICG in 245 women and reported a detection rate of 96.3% (95% CI, 81.0–99.9%) overall and 82% bilaterally. For tumors > 20 mm, the bilateral detection was 80.9%; for tumors ≤ 20 mm, the bilateral detection was 83.1% [28]. The sensitivity was 96.3%, and the NPV was 98.7% (95% CI, 93.0–100%).
- Papadia et al. (n = 60, stages IA1–IIA) found a sensitivity of 93%, a specificity of 100%, a PPV of 100%, and an NPV of 97%. The bilateral detection was 83.4%, and the overall detection was 91.7% [55]. In 22 patients (36.7%), the combination of Tc99 + blue dye was used, and in 38 patients (63.3%), −ICG was used.
- The SENTIX trial (n = 395, tumors ≤ 4 cm) reported bilateral detection in 91%, most commonly using blue dye + radiocolloid [59].
- Chiyoda et al.’s meta-analysis found unilateral SLN detection rates of 95.7–100% and bilateral rates of 80.4–90% for 99 mTc ± blue dye and ICG alone [60].
- Another meta-analysis of seven studies (589 patients with early-stage cervical cancer) found that ICG had a higher bilateral detection rate than the combined 99 mTc/blue dye method (90.3% vs. 73.5%). However, the evidence quality was low [61].

In summary, SLNB demonstrates high detection rates and low false-negative rates when bilateral mapping is achieved, particularly in tumors ≤ 2 cm and when ICG is used as a tracer. These findings validate SLNB as a reliable staging method in appropriately selected early-stage cases.

### 3.6. What Are the Most Common Anatomical Locations of SLNs in Early-Stage Cervical Cancer?

The average number of SLNs per patient ranges between 2.7 and 3.8 [51]. The most frequent locations are the external and interiliac regions (termed “typical” sites), with detection rates between 76% and 83% (Table 2) [62,63]. “Atypical” sites include paraaortic, common iliac, and internal iliac (including presacral and parametrial regions), where SLNs are found in 25–38% of cases. In 5–11%, SLNs are located exclusively in these atypical areas [51].

This table summarizes the typical and atypical locations of SLNs reported in the literature, emphasizing detection frequency and clinical relevance.

- Balaya et al. (n = 326) reported SLN detection in the interiliac or external iliac area in 83.2%, 9.2% in the common iliac area, 3.9% in the parametrium, 1.6% in the promontory area, 1.5% in the paraaortic area, and 0.5% in other areas [62]. In 10.7% of the patients, they found atypical SLN without SLN in the typical area on one or both sides and concluded that a tumor size of more than 20 mm and nulliparity increase the risk of having exclusive atypical SLN in early-stage cervical cancer [62].
- Lührs et al. (n = 145) reported bilateral obturator node detection in 69.4% (left) and 68.7% (right); external iliac nodes in 84.4% and 82.3%; common iliac nodes in 13.6% and 21.1%; and presacral nodes in 76.9% and 81.6% [64], possibly due to ICG use.
- Cibula et al. (n = 395) reported external iliac SLNs in 48% and 46% (left/right), internal iliac in 51% and 54%, and presacral nodes only on the right (6%) [59].
- Isolated SLNs in only presacral or common iliac areas were observed in 4% of cases [59].
- Ouldamer et al. presented a meta-analysis including 27 articles with 1301 patients and 3012 detected SLNs. They reported that 83.7% of the SLNs were found in classic areas of the pelvis (obturator, external iliac, and internal iliac), 6.6% in the common iliac area, 4.3% in the parametrial area, 2.0% in the paraaortic area, 1.3% in the presacral area, 0.2% in the hypogastric area, 0.07% in the inguinal area, and 0.07% in the cardinal ligament area [65].

It is important to mention that according to most authors, the obturator nodes are considered as part of the internal iliac nodes; for this reason, obturator SLNs are often not reported separately. The low detection rate of SLNs in the paraaortic area in early cervical cancer makes its search meaningless. There is evidence that metastatic LNs can present in parauterine lymphovascular tissue (PULT) and in lateral paracervical lymphatic tissue. There are many contradictions about what should be performed if SLNs are found in these areas. A survey conducted among international experts showed that 80% of them think an LN in these regions should be considered an SLN if stained with ICG; >95% think adjuvant treatment for macro- or micrometastases in this area should be conducted [66].

### 3.7. What Is the Recommended Surgical Approach When No SLNs Are Detected?

In cases where no SLNs are identified, a complete pelvic lymphadenectomy is performed. But first, re-injection of the tracer should be considered, especially if ICG was used. Maramai et al. reported bilateral detection in 184 (73.3%), unilateral detection in 57 (22.7%), and no detection in 10 (4.0%) patients at the first injection of ICG. After cervical re-injection, the bilateral detection rate increased to 94.5% (222/235), whereas unilateral detection and no detection were 5.1% (12/235) and 0.4% (1/235), respectively [67].

If SLNs are absent on only one pelvic side, dissection is limited to that side [68]. According to NCCN guidelines, SLNB alone is considered appropriate for tumors ≤ 4 cm, with the best detection rates observed in tumors ≤ 2 cm [17]. In the presence of a metastatic SLN, ESGO guidelines recommend terminating the surgical procedure [16].

### 3.8. Should SLNs Be Examined Intraoperatively (Frozen Section) or as Paraffin-Embedded Tissue Sections? Are the Results Comparable?

SLN histopathologic evaluation is crucial and may be performed via intraoperative frozen section or deferred permanent section. Frozen section carries the risk of false negatives and potential loss of tissue required for subsequent analysis. Permanent section allows for ultrastaging, which may uncover clinically significant findings missed intraoperatively.

- The Sophie Bats et al. (n = 139) trial (prospective) showed sensitivity, specificity, positive predictive, and negative predictive values for the diagnosis of macrometastases of 55.6% [95%CI: 21.2–86.3%], 100% [95%CI: 98.5–100.0%], 100% [95%CI: 47.8–100.0%], and 98.3% [95%CI: 95.8–99.5%], respectively [69].
- Slama et al. (n = 225) reported a sensitivity of 63% for macro/micrometastases and 81% for macrometastases, with an NPV of 91% [70].
- Martinez et al. (n = 225) found sensitivities of 88.9% (macro/micro) and 100% (macro) and an NPV of 98.8% [71].
- Rychlik et al. (n = 176) reported 76.9% sensitivity (macro) (95% CI, 49.7 to 91.8), 81.2% sensitivity (macro/micro) (95% CI, 57.0 to 93.4), and an NPV of 97.9% (95% CI, 93.9 to 99.3) [72].
- Sonoda et al. (n = 201) reported 100% sensitivity for macrometastases [73].
- SENTI-ENDO (n = 125) showed a sensitivity of 85.7% (95% CI, 42–99.6), an NPV of 96.8%95% CI, 83.8–99.9), and a specificity of 97.3% (95% CI, 85.8–99.9) [74].
- A meta-analysis of 14 studies (1270 patients) showed that frozen section detects 65% of nodal metastases (95% CI, 51–77%), increasing to 72% (95% CI, 60–82%) when isolated tumor cells (ITCs) are excluded [75].

To mitigate diagnostic errors, McCluggage and Cibula proposed a standardized pathology protocol for intraoperative SLN evaluation [76]. Overall, frozen section sensitivity ranged from 42.3% to 87.5% and NPV from 89.7% to 98%; excluding ITCs, the sensitivity improved to 56.4–88.9% and NPV to 91–98.8% [69,70,71,77,78].

### 3.9. What Is the Role of SLN Ultrastaging—Particularly Regarding MM and ITCs?

NCCN guidelines recommend ultrastaging of SLNs, a specialized pathological technique enabling the detection of low-volume metastases often missed by conventional histology [79]. This approach may increase metastasis detection by up to 15% [80]. According to the AJCC, nodal metastases are classified as macrometastases (>2 mm), MMs (0.2–2 mm), and ITCs (<0.2 mm) [81].

Routine lymphadenectomy specimens are not ultrastaged due to logistical and financial limitations; however, SLNs are suitable for this purpose and can lead to upstaging to FIGO IIIC if MMs or ITCs are identified [82]. Around 15–20% of histologically negative nodes may contain MMs, aligning with observed recurrence rates in node-negative patients [83].

While the prognostic significance of macrometastases is well established, the impact of MMs and ITCs remains uncertain [46]. The revised FIGO classification categorizes MMs as stage IIIC, whereas ITCs do not change staging [84]. ITCs appear not to influence oncologic outcomes or alter treatment plans [84]. For FIGO 2018 IA2–IB2 patients, MMs occur in 5–15% and ITCs in 4–7% [82,85].

- The review article by Delomenie et al. analyzed the literature up to January 2019 and reported that the available data cannot determine how to treat patients with MMs and ITCs. They expressed an opinion that small nodal disease has to be treated as a high-risk group [86].
- Cibula et al. reported a detection rate of macrometastases, MMs, and ITCs in SLN by ultrastaging in 14.7%, 10.1%, and 4.5% of patients, respectively, and established significantly reduced overall survival (OS) in patients with MMs vs. negative lymph node status (*p* < 0.001). This pattern is not observed when comparing OS in MM vs. macrometastasis [83].
- Marchiolé et al. identified MMs as an independent risk factor for recurrence in early cervical cancer and reported that MMs occur only in LVSI-positive tumors [87].
- Zaal et al. noted that survival improves with dissection of >16 nodes in patients with MMs but found no prognostic value for ITCs [88].
- Horn et al. demonstrated significantly reduced 5-year disease-free survival in patients with MMs vs. N0 (68.9% vs. 91.4%, *p* < 0.001), and the 5-year OS rate was decreased in patients with MMs vs. N0 (63.8% vs. 86.6%. But it is important to note that the included patients were IB to IIB FIGO 1988 [89].
- Guani et al. reported no impact of MMs or ITCs on recurrence-free survival [90].

Analysis of SENTICOL I and II further supports that low-volume metastases (MMs/ITCs) are associated with inferior outcomes compared to node-negative patients.

A retrospective international study involving 645 patients demonstrated that MMs impact OS similarly to macrometastases, whereas ITCs do not influence survival outcomes [71].

To date, there is no consensus on the clinical significance of low-volume metastatic disease. Consequently, the possibility that the role of ultrastaging may be overestimated should not be excluded [91].

Despite the lack of definitive data, we believe that micrometastases (MMs) should be managed with the same clinical caution as macrometastases. Multiple prospective and retrospective studies, including data from the SENTICOL trials and international multicenter analyses, have demonstrated significantly reduced disease-free and overall survival in patients with MMs compared to node-negative patients [92]. Furthermore, MMs are often associated with lymphovascular space invasion and other high-risk features. In this context, ultrastaging becomes a valuable tool for detecting clinically significant low-volume disease that may otherwise go unnoticed [93]. Until future trials provide stronger prognostic stratification, we recommend treating MMs similarly to macrometastases in early-stage cervical cancer.

### 3.10. Does Intraoperative Sentinel Lymph Node Biopsy (SLNB) Influence Oncologic Outcomes and Survival?

A meta-analysis by Chiyoda et al., including one randomized clinical trial and five observational studies, reported that SLNB alone in early-stage cervical cancer (tumors ≤ 4 cm) does not compromise disease-free or overall survival. Technetium-99 m (Tc-99m), with or without blue dye, or ICG alone may be used for SLN detection [60].

Another meta-analysis including 2226 patients across four studies with FIGO 2009 stages IA–IIA cervical cancer reported a three-year disease-free survival (DFS) of 93.1% in patients undergoing SLNB alone vs. 92.5% in those undergoing SLNB followed by pelvic lymphadenectomy (*p* = 0.773) [94].

Casper Tax et al. reviewed 47 studies involving 4130 patients with tumors ≤ 4 cm. Their analysis concluded that in patients with no suspicious preoperative or intraoperative lymph nodes and bilateral SLNs negative for metastasis after ultrastaging, the residual risk of occult nodal disease is only 0.08%. Based on this finding, the authors recommended omitting pelvic lymphadenectomy in such cases [15].

A separate meta-analysis by Ronsini et al., including 1952 patients with tumors ≤ 4 cm, directly compared oncologic outcomes between SLNB and pelvic lymphadenectomy. Their findings suggested that SLNB does not result in inferior oncologic outcomes. On the contrary, the higher rate of metastasis detection and the lower incidence of nodal recurrence observed with SLNB supported the notion that it may be oncologically equivalent to lymphadenectomy, with the added benefit of reducing surgical morbidity [95].

Notably, the SCCAN study retrospectively analyzed 1083 patients divided into two groups: one underwent pelvic lymphadenectomy alone, and the other underwent SLNB plus lymphadenectomy, with ultrastaging performed for all SLNs. SLNB led to improved detection of metastases, resulting in a lower recurrence rate (3.7% vs. 8.4%) and fewer cancer-related deaths (1.3% vs. 3.8%). Five-year disease-free survival was 96% in the SLNB group vs. 92% in the group without SLNB (95% CI, 93.5–98.5 vs. 95% CI, 90.0–94.0; *p* = 0.024). Although five-year OS was comparable (96.8% vs. 98.4%) (98.4%, 95% CI:96.8–99.9 vs. 96.8%, 95% CI, 95.4–98.2; *p* = 0.160), the rate of central pelvic recurrences was higher in the group without SLNB (4.5% vs. 1.7%) [96].

Similarly, the SENTICOL II study reported comparable 4-year disease-free survival rates between patients undergoing SLNB alone and those undergoing both SLNB and pelvic lymphadenectomy—89.5% vs. 93.1% (*p* = 0.53), respectively [97].

Taken together, the available literature indicates that SLNB does not compromise oncologic outcomes when compared to pelvic lymphadenectomy. On the contrary, SLNB may enhance the detection of micrometastases, which could lead to more frequent administration of adjuvant therapy and, potentially, improved survival (Table 3).

Table 3 shows a comparison of oncological outcomes, surgical approaches, and tracers used. DFS and OS percentages are included when specific data were available. For studies without exact figures, a dash (–) indicates that the data were not reported. Sample sizes (n) represent the number of participants, when available. The Surgical Approach column indicates the surgical approaches employed in each study. The Tracer Used column indicates the tracers utilized for sentinel lymph node biopsy.

Overall, these studies consistently show that SLNB, when properly performed and followed by ultrastaging, provides comparable oncologic outcomes to pelvic lymphadenectomy. Furthermore, it may increase micrometastasis detection and reduce overtreatment in node-negative patients.

### 3.11. What Is the Cost-Effectiveness Ratio, and How Should the Procedure Cost Be Determined, with a Focus on Pathological Assessment Activities?

Lymph node metastases are rare in early-stage cervical cancer (up to 30% in patients with stage IB carcinoma and less than 15% in patients with tumors measuring 2 cm or less) [1,2,3], which means that lymphadenectomy (LND) is often unnecessary. This fact highlights the importance of the cost-effectiveness ratio when comparing sentinel lymph node biopsy (SLNB) to pelvic lymphadenectomy in patients with early cervical cancer. Unlike other gynecological tumors, there are relatively few studies addressing this issue in cervical cancer. When conducting such analyses, it is essential to clarify what costs are considered—direct costs, expected complications, the specific disease stage, the mapping method used, etc.

A study by Brar et al. reported that SLNB using Tc99 and blue dye is a more cost-effective approach compared to pelvic LND in both the short and long term [98]. They also concluded that the use of ICG is an even more economical method for performing SLNB.

Suidan et al. categorized costs into hospital, physician, operating room, pathology (including ultrastaging), and lymphedema treatment costs [99]. They found that compared to pelvic LND, SLNB incurs lower costs and is associated with higher quality-adjusted survival, making it the most cost-effective treatment strategy for early cervical cancer.

### 3.12. Is Performing SLNB Associated with the Incidence of Early and Late Postoperative Complications?

Performing SLNB reduces the risk of complications such as nerve, major vessel, and ureter injury, less intraoperative blood loss and operative time, sensory loss, and lymphedema [100,101].

Lower limb lymphedema is one of the most disabling long-term complications related to pelvic lymphadenectomy. Its reported incidence varies widely depending on the diagnostic criteria used, with most studies citing rates between 10% and 15% [102], although some report rates as high as 40% [103]. Lymphedema negatively impacts patients’ quality of life and may lead to substantial healthcare costs [103].

SLNB may offer an alternative to pelvic LND, potentially reducing the incidence of this condition. Few studies have directly compared lymphedema rates between SLNB and pelvic LND in cervical cancer.

Reported lymphedema incidence after cervical cancer treatment varies broadly from 0% to 69%, depending on diagnostic methods [104,105]. Studies using subjective criteria (questionnaires and self-assessment) tend to report higher rates than those using objective measures (clinical diagnosis, diagnostic tests, and validated international classifications) [106]. Incidence ranges from 7% to 69% in studies with subjective criteria [107], and from 0% to 58% in studies with objective criteria [104,105].

The SENTIREC study reported early lymphedema rates of 5.6% (95% CI, 2.1–11.8%) in patients who underwent SLNB alone and 32.3% (95% CI, 22.9–42.7%) in those who had SLNB plus LND [108]. Niikura et al. found a significantly lower lymphedema rate in patients who underwent SLNB only compared to those with LND (8.7% vs. 42%) (*p* = 0.03) [109]. SENTICOL II reported lymphedema rates of 31.4% vs. 51.5% (51.5%; *p* = 0.0046), and fewer early postoperative neurological complications (7.8% vs. 20.6%, *p* = 0.01, respectively) in SLNB vs. LND groups, respectively [30]. Lennox et al. documented shorter operative time (2 vs. 2.8 h, *p* < 0.001), less intraoperative blood loss (100 vs. 500 mL, *p* < 0.001), fewer blood transfusions (0% vs. 23%, *p* < 0.001), lower postoperative infection rates (0% vs. 11%, *p* = 0.001), and shorter hospital stays following SLNB [110]. Gianoni et al. showed trends toward a better quality of life and less leg heaviness and fatigue when pelvic lymphadenectomy was avoided [111].

A prospective analysis of lymphedema risk after SLNB showed cumulative lymphedema rates at 24 months of 17.3% for mild, 9.2% for moderate, and 0.7% for severe forms, with a median time to onset of nine months [112]. The authors concluded that SLNB in cervical cancer surgery does not eliminate the risk of mild to moderate lymphedema, which develops regardless of the number of sentinel lymph nodes removed.

SLNB can lead to certain complications, but there are no studies in the literature specifically focusing on the complications of SLNB in early cervical cancer.

These data confirm that SLNB is associated with significantly fewer postoperative complications, particularly in relation to lymphedema and infection. Nevertheless, SLNB is not entirely risk-free, and long-term surveillance for mild to moderate lymphedema remains important.

### 3.13. Are There Risk Factors, Such as High BMI, Prior Conization, or Tumor Size, That Contraindicate Intraoperative SLNB?

Unlike endometrial carcinoma, where risk factors for unsuccessful SLNB are well documented, these remain unclear in cervical cancer due to limited studies. One study involving 405 patients using a combined mapping method identified age ≥ 70 years (*p* = 0.004), BMI > 30 kg/m^2^ (*p* = 0.048), and tumor size ≥ 20 mm (*p* = 0.048) as factors associated with failure to detect bilateral sentinel lymph nodes [113]. The same study noted that success rates depend on surgical team experience and that minimally invasive approaches tend to improve successful SLN mapping compared to open surgery (87.9% vs. 80.6%).

Kiss et al. reported that larger tumors (>4 cm), deep stromal invasion, and prior conization negatively impacted detection rates of SLN. However, their cohort only included 42 patients with FIGO 2018 stages IA1–IIA1, and they used methylene blue alone or combined with 99 mTc. In 27 patients (64.3%), blue dye only was used, and the overall detection and bilateral detection rates were 70.4% and 40.4%, respectively [114]. This compromised the results of their study.

Another study found lymphovascular space invasion (LVSI) to be the only factor that impeded SLN detection (41.5% vs. 90.9%, *p* < 0.001), while other factors, such as tumor diameter, growth type, histological grade, deep stromal invasion, and neoadjuvant chemotherapy, showed no significant impacts [115].

An interesting situation occurs when SLNB must be performed after conization. There is not much data in the literature on this topic. In 18 patients after conization, Kato et al. reported 100% (95% CI, 0.815–1.00) and 72.2% (95% CI, 0.465–0.90) detection rates, unilaterally and bilaterally, respectively; the average number of the detected SLN was 2.4; the negative and positive predictive value was 100% (95% CI, 0.158–1.00), and they did not find statistical significance when compared to non-conization patients [116].

### 3.14. Prespectives

In recent years, artificial intelligence and machine learning have been increasingly embedded in medicine. They are also beginning to find their place in the detection and pathoanatomical examination of SLNs in cervical cancer. Various systems are under development to improve the detection of metastatic SLNs using machine learning and ultrasound-based radiomics [117] or magnetic resonance imaging radiomics [118,119]. Another possible application of deep learning algorithms is the evaluation of slices in ultrastaging of SLNs, which is an expensive, slow, and laborious process. These algorithms can help pathologists efficiently evaluate serial sections for metastases, reducing workload and costs while increasing accuracy [120].

#### Limitations

While this review incorporates numerous prospective trials and meta-analyses, several limitations must be acknowledged. First, most of the included studies are heterogeneous in terms of patient selection, tracer use, surgical approach, and pathological evaluation, which makes direct comparison challenging. Second, a substantial portion of the data derives from retrospective or single-institution studies, particularly in areas concerning ultrastaging and management of micrometastases. Third, although SLNB shows promise in tumors ≤ 4 cm, the strongest evidence exists for tumors < 2 cm. Finally, long-term data on survival outcomes and recurrence patterns following SLNB alone remain limited. These limitations underscore the need for additional large-scale, multicenter randomized trials with standardized protocols.

**Figure 2:**
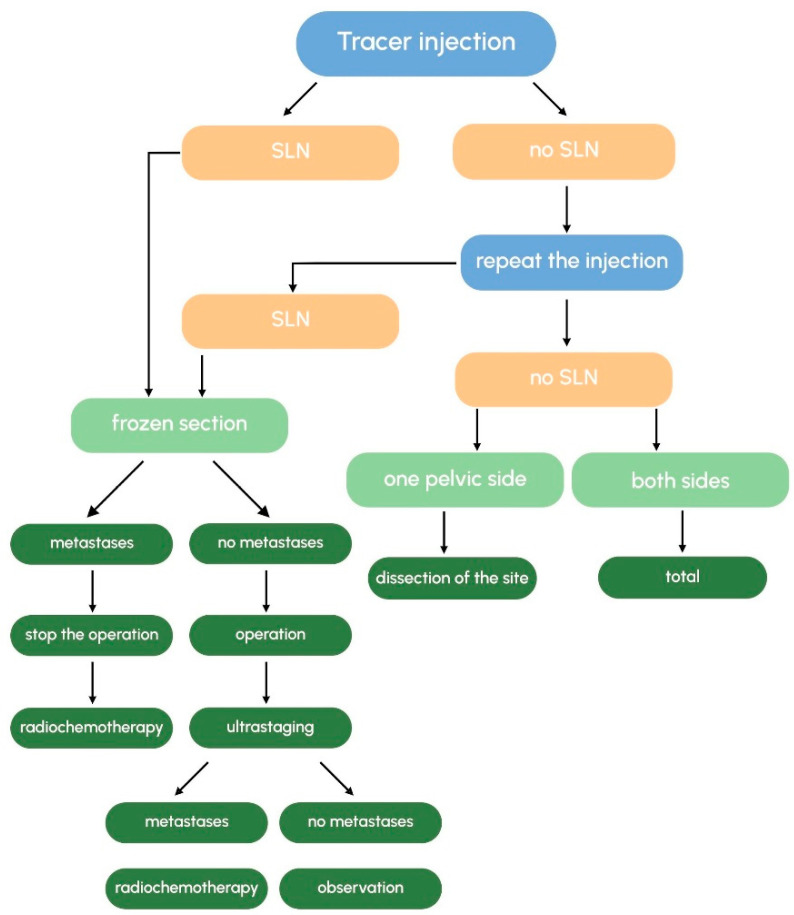
Tracer injection pathway.

Based on the available information, we propose an algorithm for performing SLNB in patients with early cervical cancer (Figure 2).

## 4. Conclusions

SLNB has emerged as a reliable and minimally invasive technique for lymph node assessment in early-stage cervical cancer. Accumulating evidence from prospective trials and meta-analyses demonstrates that SLNB offers high detection rates, sensitivity, and negative predictive value, particularly when bilateral mapping is achieved and advanced tracer techniques are employed. Compared to systematic pelvic lymphadenectomy, SLNB significantly reduces surgical morbidity, including the risk of lower limb lymphedema, without compromising oncologic outcomes, such as disease-free and overall survival.

Current guidelines support the use of SLNB as a standard approach for selected patients with early-stage disease, especially those with tumors less than 4 cm and no evidence of lymph node metastasis on imaging. The technique also facilitates ultrastaging, enabling the detection of low-volume metastatic disease that may influence adjuvant treatment decisions.

Despite these advantages, certain limitations remain, including the need for further standardization of surgical and pathological protocols and the identification of patient- or tumor-related factors that may affect mapping success. Ongoing research is warranted to refine patient selection criteria and optimize the clinical application of SLNB in cervical cancer. SLNB represents a paradigm shift in the surgical management of early cervical cancer, offering accurate staging with reduced morbidity and supporting a more individualized, evidence-based approach to patient care.

## Figures and Tables

**Table 1:** SLNB detection rates, sensitivities, and false negative rates from major studies.

Study	Tracer Used	Detection Rate (Overall/Bilateral)	Sensitivity (%)	False Negative Rate (%)	NPV (%)	Surgical Approach
SENTICOL I [13]	Blue dye + 99 mTc	97.8%/76.5%	92	1.4	98.2	Laparoscopy
SENTIREC [28]	ICG	96.3%/82%	96.3	Not reported	98.7	Laparoscopy
Kim et al. [57]	ICG	100%/85.4%	71.4 (100% < 2 cm)	23.1	92.4	Robot-assisted laparoscopic
Papadia et al. [55]	Tc99 + dye/ICG	91.7%/83.4%	93	Not reported	97	Laparoscopy
Dostalek et al. [58]	Blue dye + 99 mTc	93%/80%	93–96	0–1.6	Not reported	Laparoscopy
SENTIX [59]	Blue dye + radiocolloid	Not reported/91%	Not reported	Not reported	Not reported	Laparoscopy
Chiyoda et al. (meta-analysis) [60]	ICG or 99 mTc ± dye	95.7–100%/80.4–90%	Varies	Varies	Varies	Mixed (all approaches)

**Table 2:** Common anatomical locations of sentinel lymph nodes (SLNs) in early-stage cervical cancer.

Anatomical Site	Detection Frequency (%)	Notes
External/interiliac area	76–83%	Most common (“typical”) location
Obturator nodes	Often grouped with internal iliac nodes	Not always reported separately
Internal iliac area	48–54%	May include presacral area
Common iliac area	6.6–21.1%	Considered “atypical”
Parametrium	3.9–4.3%	Challenging to access
Presacral area	1.3–6%	Detected more frequently with ICG
Paraaortic area	1.5–2.0%	Rare; detection often not clinically relevant
Hypogastric, inguinal, cardinal ligament	<0.5%	Extremely rare sites

**Table 3:** Comparative oncological outcomes: SLNB vs. PLND.

Study	Population/FIGO Stage (Year of Classification)	n	Procedure	DFS (%)	OS (%)	Surgical Approach	Tracer Used
Chiyoda et al. [60] (meta-analysis, 2022)	Early-stage cervical cancer ≤ 4 cm (FIGO 2009)	Not found	SLNB vs. pelvic LND	No significant difference	No significant difference	Laparoscopy, Laparotomy	Tc-99 m ± blue dye or ICG
Mauro et al. [94] (2023)	FIGO IA–IIA (FIGO 2018)	2226	SLNB vs. SLNB + LND	93.1 vs. 92.5 (3-yr DFS)	Not reported	Laparoscopy, Laparotomy	Tc-99 m, blue dye, ICG
Casper Tax et al. [15] (2015)	FIGO IA–IB1 ≤ 4 cm (FIGO 2009)	4130	SLNB (bilateral negative) only	–	–	Laparoscopy, Laparotomy	ICG, Tc-99 m ± blue dye
Ronsini et al. [95] (2022)	FIGO IA–IB1 ≤ 4 cm (FIGO 2009)	1952	SLNB vs. LND	–	–	Laparoscopy, Laparotomy	Tc-99 m ± blue dye, ICG
SCCAN study [96] (2024)	Early-stage (FIGO 2009 IA–IB)	1083	SLNB + LND vs. LND only	96 vs. 92 (5-yr DFS)	96.8 vs. 98.4 (5-yr OS)	Laparoscopy, Laparotomy	ICG, Tc-99 m ± blue dye
SENTICOL II [97] (prospective 2021)	Early-stage (FIGO 2009 IA–IB)	206	SLNB vs. SLNB + LND	89.5 vs. 93.1 (4-yr DFS)	Not reported	Laparoscopy, Robotic	ICG ± Tc-99 m

## Data Availability

The authors declare that all related data are available from the corresponding author upon reasonable request.

## References

[1] World Health Organization Cervical Cancer. https://www.who.int/news-room/fact-sheets/detail/cervical-cancer.

[2] Bhatla N., Aoki D., Sharma D.N., Sankaranarayanan R. (2018). Cancer of the cervix uteri. Int. J. Gynaecol. Obstet..

[3] Guida F., Kidman R., Ferlay J., Schüz J., Soerjomataram I., Kithaka B., Ginsburg O., Mailhot Vega R.B., Galukande M., Parham G. (2022). Global and regional estimates of orphans attributed to maternal cancer mortality in 2020. Nat. Med..

[4] ESGO Cervical Cancer. Pocket Guidelines. https://www.esgo.org/media/2019/03/Pocket-Guidelines_Cervical-cancer_June2023.pdf.

[5] National Comprehensive Cancer Network. https://www.nccn.org/professionals/physician_gls/pdf/cervical.pdf.

[6] Ferrandina G., Pedone Anchora L., Gallotta V., Fagotti A., Vizza E., Chiantera V., De Iaco P., Ercoli A., Corrado G., Bottoni C. (2017). Can we define the risk of lymph node metastasis in early-stage cervical cancer patients? A large-scale, Retrospective Study. Ann. Surg. Oncol..

[7] Cabanas R.M. (1977). An approach for the treatment of penile carcinoma. Cancer.

[8] Lyman G.H., Giuliano A.E., Somerfield M.R., Benson A.B., Bodurka D.C., Burstein H.J., Cochran A.J., Cody H.S., Edge S.B., Galper S. (2005). American Society of Clinical Oncology guideline recommendations for sentinel lymph node biopsy in early-stage breast cancer. J. Clin. Oncol..

[9] Morton D.L., Thompson J.F., Cochran A.J., Mozzillo N., Nieweg O.E., Roses D.F., Hoekstra H.J., Karakousis C.P., Puleo C.A., Coventry B.J. (2014). Final trial report of sentinel-node biopsy versus nodal observation in melanoma. N. Engl. J. Med..

[10] Cibula D., Abu-Rustum N.R., Dusek L., Slama J., Zikán M., Zaal A., Sevcik L., Kenter G., Querleu D., Jach R. (2012). Bilateral ultrastaging of sentinel lymph node in cervical cancer: Lowering the falsenegative rate and improving the detection of micrometastasis. Gynecol. Oncol..

[11] Altgassen C., Hertel H., Brandstädt A., Köhler C., Dürst M., Schneider A. (2008). Multicenter validation study of the sentinel lymph node concept in cervical cancer: AGO Study Group. J. Clin. Oncol..

[12] Diaz J.P., Gemignani M.L., Pandit-Taskar N., Park K.J., Murray M.P., Chi D.S., Sonoda Y., Barakat R.R., Abu-Rustum N.R. (2011). Sentinel lymph node biopsy in the management of early-stage cervical carcinoma. Gynecol. Oncol..

[13] Lécuru F., Mathevet P., Querleu D., Leblanc E., Morice P., Daraï E., Marret H., Magaud L., Gillaizeau F., Chatellier G. (2011). Bilateral negative sentinel nodes accurately predict absence of lymph node metastasis in early cervical cancer: Results of the SENTICOL study. J. Clin. Oncol..

[14] Du X.-L., Sheng X.-G., Jiang T., Li Q.-S., Yu H., Pan C.-X., Lu C.-H., Wang C., Song Q.-Q. (2011). Sentinel lymph node biopsy as guidance for radical trachelectomy in young patients with early stage cervical cancer. BMC Cancer.

[15] Tax C., Rovers M.M., de Graaf C., Zusterzeel P.L., Bekkers R.L. (2015). The sentinel node procedure in early stage cervical cancer, taking the next step; a diagnosticreview. Gynecol. Oncol..

[16] Cibula D., Pötter R., Planchamp F., Avall-Lundqvist E., Fischerova D., Meder C.H., Köhler C., Landoni F., Lax S., Lindegaard J.C. (2018). The European Society of Gynaecological Oncology/European Society for Radiotherapy and Oncology/European Society of Pathology guidelines for the management of patients with cervical cancer. Radiother. Oncol..

[17] National Comprehensive Cancer Network (2025). NCCN Clinical Practice Guidelines in Oncology: Cervical Cancer, Version 2.2025.

[18] Dargent D., Martin X., Mathevet P. (2000). Laparoscopic assessment of the sentinel lymph node in early stage cervical cancer. Gynecol. Oncol..

[19] Medl M., Peters-Engl C., Schütz P., Vesely M., Sevelda P. (2000). First report of lymphatic mapping with isosulfan blue dye and sentinel node biopsy in cervical cancer. Anticancer Res..

[20] O’Boyle J.D., Coleman R.L., Bernstein S.G., Lifshitz S., Muller C.Y., Miller D.S. (2000). Intraoperative lymphatic mapping in cervix cancer patients undergoing radical hysterectomy: A pilot study. Gynecol. Oncol..

[21] Kamprath S., Possover M., Schneider A. (2000). Laparoscopic sentinel lymph node detection in patients with cervical cancer. Am. J. Obstet. Gynecol..

[22] Ramirez P.T., Levenback C. (2001). Sentinel nodes in gynecologic malignancies. Curr. Opin. Oncol..

[23] Verheijen R.H., Pijpers R., van Diest P.J., Burger C.W., Buist M.R., Kenemans P. (2000). Sentinel node detection in cervical cancer. Obstet. Gynecol..

[24] Malur S., Krause N., Köhler C., Schneider A. (2001). Sentinel lymph node detection in patients with cervical cancer. Gynecol. Oncol..

[25] van Dam P.A., Hauspy J., Vanderheyden T., Sonnemans H., Spaepen A., Eggenstein G., Dirix L., Verkinderen L. (2003). Intraoperative sentinel node identification with Technetium-99m-labeled nanocolloid in patients with cancer of the uterine cervix: A feasibility study. Int. J. Gynecol. Cancer.

[26] Levenback C., Coleman R.L., Burke T.W., Lin W.M., Erdman W., Deavers M., Delpassand E.S. (2002). Lymphatic mapping and sentinel node identification in patients with cervix cancer undergoing radical hysterectomy and pelvic lymphadenectomy. J. Clin. Oncol..

[27] Salvo G., Ramirez P.T., Levenback C.F., Munsell M.F., Euscher E.D., Soliman P.T., Frumovitz M. (2017). Sensitivity and negative predictive value for sentinel lymph node biopsy in women with early-stage cervical cancer. Gynecol. Oncol..

[28] Sponholtz S.E., Mogensen O., Hildebrandt M.G., Schledermann D., Parner E., Markauskas A., Frøding L.P., Fuglsang K., Vilstrup M.H., Bjørnholt S.M. (2021). Sentinel lymph node mapping in early-stage cervical cancer—A national prospective multicenter study (SENTIREC trial). Gynecol. Oncol..

[29] Abu-Rustum N.R., Yashar C.M., Bean S., Bradley K., Campos S.M., Chon H.S., Chu C., Cohn D., Crispens M.A., Damast S. (2020). NCCN Guidelines Insights: Cervical Cancer, Version 1.2020. J. Natl. Compr. Cancer Netw..

[30] Mathevet P., Lécuru F., Uzan C., Boutitie F., Magaud L., Guyon F., Querleu D., Fourchotte V., Baron M., Bats A.S. (2021). Sentinel lymph node biopsy and morbidity outcomes in early cervical cancer: Results of a multicentre randomised trial (SENTICOL-2). Eur. J. Cancer..

[31] Lecuru F.R., McCormack M., Hillemanns P., Anota A., Leitao M., Mathevet P., Zweemer R., Fujiwara K., Zanagnolo V., Zahl Eriksson A.G. (2019). SENTICOL III: An international validation study of sentinel node biopsy in early cervical cancer. A GINECO, ENGOT, GCIG and multicenter study. Int. J. Gynecol. Cancer.

[32] Cibula D., Kuzel D., Sláma J., Fischerova D., Dundr P., Freitag P., Zikán M., Pavlista D., Tomancova V. (2009). Sentinel node (SLN) biopsy in the management of locally advanced cervical cancer. Gynecol. Oncol..

[33] Slama J., Dundr P., Dusek L., Fischerova D., Pinkavova I., Zikan M., Vrzackova P., Kojanova M., Cibula D. (2012). Sentinel lymph node status in patients with locally advanced cervical cancers and impact of neoadjuvant chemotherapy. Gynecol. Oncol..

[34] Sláma J., Zikán M., Fischerová D., Kocián R., Germanová A., Frühauf F., Cibula D. (2016). Contribution of sentinel lymph-node biopsy to treatment of locally advanced stages of cervical cancers. Ceska Gynekol..

[35] Barranger E., Coutant C., Cortez A., Uzan S., Darai E. (2005). Sentinel node biopsy is reliable in early-stage cervical cancer but not in locally advanced disease. Ann. Oncol..

[36] Daraï E., Lavoué V., Rouzier R., Coutant C., Barranger E., Bats A.S. (2007). Contribution of the sentinel node procedure to tailoring the radicality of hysterectomy for cervical cancer. Gynecol. Oncol..

[37] Lavoué V., Bats A.S., Rouzier R., Coutant C., Barranger E., Daraï E. (2007). Sentinel lymph node procedure followed by laparoscopic pelvic and paraaortic lymphadenectomy in women with IB2-II cervical cancer. Ann. Surg. Oncol..

[38] Bray F., Loos A.H., McCarron P., Weiderpass E., Arbyn M., Møller H., Hakama M., Parkin D.M. (2005). Trends in cervical squamous cell carcinoma incidence in 13 European countries: Changing risk and the effects of screening. Cancer Epidemiol. Biomark. Prev..

[39] Wang S.S., Sherman M.E., Hildesheim A., Lacey J.V., Devesa S. (2004). Cervical adenocarcinoma and squamous cell carcinoma incidence trends among white women and black women in the United States for 1976–2000. Cancer.

[40] Castellsague X., Diaz M., de Sanjose S., Muñoz N., Herrero R., Franceschi S., Peeling R.W., Ashley R., Smith J.S., Snijders P.J.F. (2006). Worldwide human papillomavirus etiology of cervical adenocarcinoma and its cofactors: Implications for screening and prevention. J. Natl. Cancer Inst..

[41] Sasieni P., Castanon A., Cuzick J. (2009). Screening and adenocarcinoma of the cervix. Int. J. Cancer.

[42] Stolnicu S., Barsan I., Hoang L., Patel P., Terinte C., Pesci A., Aviel-Ronen S., Kiyokawa T., Alvarado-Cabrero I., Pike M.C. (2018). International Endocervical Adenocarcinoma Criteria and Classification (IECC): A New Pathogenetic Classification for Invasive Adenocarcinomas of the Endocervix. Am. J. Surg. Pathol..

[43] Balaya V., Guani B., Pache B., Durand Y.G., Bonsang-Kitzis H., Ngô C., Mathevet P., Lécuru F. (2021). Sentinel lymph node in cervical cancer: Time to move forward. Chin. Clin. Oncol..

[44] Ramirez P.T., Frumovitz M., Pareja R., Lopez A., Vieira M., Ribeiro M., Buda A., Yan X., Shuzhong Y., Chetty N. (2018). Minimally Invasive versus Abdominal Radical Hysterectomy for Cervical Cancer. N. Engl. J. Med..

[45] Wang X.J., Fang F., Li Y.F. (2015). Sentinel-lymph-node procedures in early stage cervical cancer: A systematic review and meta-analysis. Med. Oncol..

[46] Margioula-Siarkou C., Almperis A., Gullo G., Almperi E.A., Margioula-Siarkou G., Nixarlidou E., Mponiou K., Papakotoulas P., Sardeli C., Guyon F. (2023). Sentinel Lymph Node Staging in Early-Stage Cervical Cancer: A Comprehensive Review. J. Clin. Med..

[47] Frumovitz M., Plante M., Lee P.S., Sandadi S., Lilja J.F., Escobar P.F., Gien L.T., Urbauer D.L., Abu-Rustum N.R. (2018). Near-infrared fluorescence for detection of sentinel lymph nodes in women with cervical and uterine cancers (FILM): A randomised, phase 3, multicentre, non-inferiority trial. Lancet Oncol..

[48] Ruscito I., Gasparri M.L., Braicu E.I., Bellati F., Raio L., Sehouli J., Mueller M.D., Panici P.B., Papadia A. (2016). Sentinel Node Mapping in Cervical and Endometrial Cancer: Indocyanine Green Versus Other Conventional Dyes-A Meta-Analysis. Ann. Surg. Oncol..

[49] Zhang X., Bao B., Wang S., Yi M., Jiang L., Fang X. (2021). Sentinel lymph node biopsy in early stage cervical cancer: A meta-analysis. Cancer Med..

[50] Wang L., Liu S., Xu T., Yuan L., Yang X. (2021). Sentinel lymph node mapping in early-stage cervical cancer: Meta-analysis. Medicine.

[51] Smits A., Ten Eikelder M., Dhanis J., Moore W., Blake D., Zusterzeel P., Kucukmetin A., Ratnavelu N., Rundle S. (2023). Finding the sentinel lymph node in early cervical cancer: When is unusual not uncommon?. Gynecol. Oncol..

[52] Hricak H., Gatsonis C., Chi D.S., Amendola M.A., Brandt K., Schwartz L.H., Koelliker S., Siegelman E.S., Brown J.J., McGhee R.B. (2005). Role of Imaging in Pretreatment Evaluation of Early Invasive Cervical Cancer: Results of the Intergroup Study American College of Radiology Imaging Network 6651-Gynecologic Oncology Group 183. J. Clin. Oncol..

[53] Kodama J., Mizutani Y., Hongo A., Yoshinouchi M., Kudo T., Okuda H. (2002). Optimal Surgery and Diagnostic Approach of Stage IA2 Squamous Cell Carcinoma of the Cervix. Eur. J. Obstet. Gynecol. Reprod. Biol..

[54] Selman T.J., Mann C., Zamora J., Appleyard T.-L., Khan K. (2008). Diagnostic Accuracy of Tests for Lymph Node Status in Primary Cervical Cancer: A Systematic Review and Meta-Analysis. Can. Med. Assoc. J..

[55] Papadia A., Gasparri M.L., Genoud S., Bernd K., Mueller M.D. (2017). The Combination of Preoperative PET/CT and Sentinel Lymph Node Biopsy in the Surgical Management of Early-Stage Cervical Cancer. J. Cancer Res. Clin. Oncol..

[56] Tanaka T., Sasaki S., Tsuchihashi H., Terai Y., Yamamoto K., Yamada T., Ohmichi M. (2018). Which Is Better for Predicting Pelvic Lymph Node Metastases in Patients with Cervical Cancer: Fluorodeoxyglucose-Positron Emission Tomography/Computed Tomography or a Sentinel Node Biopsy? A Retrospective Observational Study. Medicine.

[57] Kim J.H., Kim D.Y., Suh D.S., Kim J.H., Kim Y.M., Kim Y.T., Nam J.H. (2018). The efficacy of sentinel lymph node mapping with indocyanine green in cervical cancer. World J. Surg. Oncol..

[58] Dostálek L., Zikan M., Fischerova D., Kocian R., Germanova A., Frühauf F., Dusek L., Slama J., Dundr P., Nemejcova K. (2018). SLN biopsy in cervical cancer patients with tumors larger than 2 cm and 4 cm. Gynecol. Oncol..

[59] Cibula D., Kocian R., Plaikner A., Jarkovsky J., Klat J., Zapardiel I., Pilka R., Torne A., Sehnal B., Ostojich M. (2020). Sentinel lymph node mapping and intraoperative assessment in a prospective, international, multicentre, observational trial of patients with cervical cancer: The SENTIX trial. Eur. J. Cancer..

[60] Chiyoda T., Yoshihara K., Kagabu M., Nagase S., Katabuchi H., Mikami M., Tabata T., Hirashima Y., Kobayashi Y., Kaneuchi M. (2022). Sentinel node navigation surgery in cervical cancer: A systematic review and metaanalysis. Int. J. Clin. Oncol..

[61] Baeten I.G.T., Hoogendam J.P., Jeremiasse B., Braat A.J.A.T., Veldhuis W.B., Jonges G.N., Jürgenliemk-Schulz I.M., van Gils C.H., Zweemer R.P., Gerestein C.G. (2022). Indocyanine green versus technetium-99m with blue dye for sentinel lymph node detection in early-stage cervical cancer: A systematic review and meta-analysis. Cancer Rep..

[62] Balaya V., Mathevet P., Magaud L., Bonsang-Kitzis H., Delomenie M., Montero Macias R., Ngô C., Bats A.S., Lécuru F. (2019). Predictive factors of unexpected lymphatic drainage pathways in early-stage cervical cancer. Gynecol. Oncol..

[63] Marnitz S., Köhler C., Bongardt S., Braig U., Hertel H., Schneider A. (2006). German Association of Gynecologic Oncologists (AGO). Topographic distribution of sentinel lymph nodes in patients with cervical cancer. Gynecol. Oncol..

[64] Lührs O., Bollino M., Ekdahl L., Lönnerfors C., Geppert B., Persson J. (2022). Similar distribution of pelvic sentinel lymph nodes and nodal metastases in cervical and endometrial cancer. A prospective study based on lymphatic anatomy. Gynecol. Oncol..

[65] Ouldamer L., Marret H., Acker O., Barillot I., Body G. (2012). Unusual localizations of sentinel lymph nodes in early stage cervical cancer: A review. Surg. Oncol..

[66] Pavone M., Bizzarri N., Rychlik A., Persson J., Fagotti A., Fanfani F., Scambia G., Querleu D. (2025). In-transit metastatic lymph nodes in cervical cancer: A new staging and therapeutic concept. Eur. J. Obstet. Gynecol. Reprod. Biol..

[67] Maramai M., Achilarre M.T., Aloisi A., Betella I., Bogliolo S., Garbi A., Maruccio M., Quatrale C., Aletti G.D., Mariani A. (2021). Cervical re-injection of indocyanine green to improve sentinel lymph node detection in endometrial cancer. Gynecol. Oncol..

[68] Cormier B., Diaz J.P., Shih K., Sampson R.M., Sonoda Y., Park K.J., Alektiar K., Chi D.S., Barakat R.R., Abu-Rustum N.R. (2011). Establishing a sentinel lymph node mapping algorithm for the treatment of early cervical cancer. Gynecol. Oncol..

[69] Bats A.-S., Buénerd A., Querleu D., Leblanc E., Daraï E., Morice P., Marret H., Gillaizeau F., Mathevet P., Lécuru F. (2011). Diagnostic value of intraoperative examination of sentinel lymph node in early cervical cancer: A prospective, multicenter study. Gynecol. Oncol..

[70] Slama J., Dundr P., Dusek L., Cibula D. (2013). High false negative rate of frozen section examination of sentinel lymph nodes in patients with cervical cancer. Gynecol. Oncol.

[71] Martínez A., Mery E., Filleron T., Boileau L., Ferron G., Querleu D. (2013). Accuracy of intraoperative pathological examination of SLN in cervical cancer. Gynecol. Oncol..

[72] Rychlik A., Angeles M.A., Migliorelli F., Croce S., Mery E., Martinez A., Ferron G., Guyon F., Querleu D. (2020). Frozen section examination of sentinel lymph nodes can be used as a decisional tool in the surgical management of early cervical cancer. Int. J. Gynecol. Cancer.

[73] Sonoda K., Yahata H., Okugawa K., Kaneki E., Ohgami T., Yasunaga M., Baba S., Oda Y., Honda H., Kato K. (2018). Value of intraoperative cytological and pathological sentinel lymph node diagnosis in fertility-sparing trachelectomy for early-stage cervical cancer. Oncology.

[74] Ballester M., Dubernard G., Bats A.-S., Heitz D., Mathevet P., Marret H., Querleu D., Golfier F., Leblanc E., Rouzier R. (2012). Comparison of diagnostic accuracy of frozen section with imprint cytology for intraoperative examination of sentinel lymph node in early-stage endometrial cancer: Results of Senti-Endo study. Ann. Surg. Oncol..

[75] Agustí N., Viveros-Carreño D., Mora-Soto N., Ramírez P.T., Rauh-Hain A., Wu C.F., Rodríguez J., Grillo-Ardila C.F., Salazar C., Jorgensen K. (2023). Diagnostic accuracy of sentinel lymph node frozen section analysis in patients with early-stage cervical cancer: A systematic review and meta-analysis. Gynecol. Oncol..

[76] Cibula D., McCluggage W.G. (2019). Sentinel lymph node (SLN) concept in cervical cancer: Current limitations and unanswered questions. Gynecol. Oncol..

[77] Roy M., Bouchard-Fortier G., Popa I., Grégoire J., Renaud M.C., Têtu B., Plante M. (2011). Value of sentinel node mapping in cancer of the cervix. Gynecol. Oncol..

[78] Balaya V., Guani B., Benoit L., Magaud L., Bonsang-Kitzis H., Ngô C., Le Frère-Belda M.A., Mathevet P., Lécuru F. (2020). Diagnostic value of frozen section examination of sentinel lymph nodes in early-stage cervical cancer at the time of ultrastaging. Gynecol. Oncol..

[79] Koh W.-J., Abu-Rustum N.R., Bean S., Bradley K., Campos S.M., Cho K.R., Chon H.S., Chu C., Clark R., Cohn D. (2019). Cervical Cancer, Version 3.2019, NCCN Clinical Practice Guidelines in Oncology. J. Natl. Compr. Canc Netw..

[80] Gortzak-Uzan L., Jimenez W., Nofech-Mozes S., Ismiil N., Khalifa M.A., Dubé V., Rosen B., Murphy J., Laframboise S., Covens A. (2010). Sentinel lymph node biopsy vs. pelvic lymphadenectomy in early stage cervical cancer: Is it time to change the gold standard?. Gynecol Oncol..

[81] Edge S.B., Compton C.C. (2010). The American Joint Committee on Cancer: The 7th Edition of the AJCC Cancer Staging Manual and the Future of TNM. Ann. Surg. Oncol..

[82] Kim C.H., Soslow R.A., Park K.J., Barber E.L., Khoury-Collado F., Barlin J.N., Sonoda Y., Hensley M.L., Barakat R.R., Abu-Rustum N.R. (2013). Pathologic Ultrastaging Improves Micrometastasis Detection in Sentinel Lymph Nodes during Endometrial Cancer Staging. Int. J. Gynecol. Cancer.

[83] Cibula D., Abu-Rustum N.R., Dusek L., Zikán M., Zaal A., Sevcik L., Kenter G.G., Querleu D., Jach R., Bats A.S. (2012). Prognostic significance of low volume sentinel lymph node disease in early-stage cervicalcancer. Gynecol. Oncol..

[84] Bhatla N., Berek J.S., Cuello Fredes M., Denny L.A., Grenman S., Karunaratne K., Kehoe S.T., Konishi I., Olawaiye A.B., Prat J. (2019). Revised FIGO staging for carcinoma of the cervix uteri. Int. J. Gynaecol. Obstet..

[85] Lentz S.E., Muderspach L.I., Felix J.C., Ye W., Groshen S., Amezcua C.A. (2004). Identification of micrometastases in histologically negative lymph nodes of earlystage cervical cancer patients. Obstet. Gynecol..

[86] Delomenie M., Bonsang-Kitzis H., Bats A.S., Ngo C., Balaya V., Xuan H.T.N., Koual M., Mathevet P., Lecuru F. (2019). The clinical implication of lymph nodes micrometastases and isolated tumor cells in patients with cervical cancer: A systematic review. Eur. J. Obstet. Gynecol. Reprod. Biol..

[87] Marchiolé P., Buénerd A., Benchaib M., Nezhat K., Dargent D., Mathevet P. (2005). Clinical Significance of Lympho Vascular Space Involvement and Lymph Node Micrometastases in Early-Stage Cervical Cancer: A Retrospective Case-Control SurgicoPathological Study. Gynecol. Oncol..

[88] Zaal A., Zweemer R.P., Zikán M., Dusek L., Querleu D., Lécuru F., Bats A.-S., Jach R., Sevcik L., Graf P. (2014). Pelvic Lymphadenectomy Improves Survival in Patients with Cervical Cancer with Low-Volume Disease in the Sentinel Node: A Retrospective Multicenter Cohort Study. Int. J. Gynecol. Cancer.

[89] Horn L.C., Hentschel B., Fischer U., Peter D., Bilek K. (2008). Detection of micrometastases in pelvic lymph nodes in patients with carcinoma of the cervix uteri using step sectioning: Frequency, topographic distribution and prognostic impact. Gynecol. Oncol..

[90] Guani B., Dorez M., Magaud L., Buenerd A., Lecuru F., Mathevet P. (2019). Impact of Micrometastasis or Isolated Tumor Cells on Recurrence and Survival in Patients with Early Cervical Cancer: SENTICOL Trial. Int. J. Gynecol. Cancer.

[91] Guani B., Balaya V., Magaud L., Lecuru F., Mathevet P. (2020). The Clinical Impact of Low-Volume Lymph Nodal Metastases in Early-Stage Cervical Cancer: The Senticol 1 and Senticol 2 Trials. Cancers.

[92] Nica A., Gien L.T., Ferguson S.E., Covens A. (2020). Does small volume metastatic lymph node disease affect long-term prognosis in early cervical cancer?. Int. J. Gynecol. Cancer.

[93] Colpaert C., Jacomen G., Van de Vijver K., Baldewijns M., Van Rompuy A.-S., Bourgain C., Noël J.-C. (2019). Ultrastaging of sentinel lymph nodes in gynecological cancer: Repeating the story of breast cancer? Letter to the editor, Reply to Cibula D, McCluggage WG. Sentinel lymph node (SLN) concept in cervical cancer: Current limitations and unanswered questions. Gynecol Oncol 2019;152:202–7. Gynecol. Oncol. Rep..

[94] Mauro J., Viveros-Carreño D., Vizzielli G., De Ponti E., Fanfani F., Ramirez P.T., Buda A. (2023). Survival after sentinel node biopsy alone in early-stage cervical cancer: A systematic review. Int. J. Gynecol. Cancer.

[95] Ronsini C., De Franciscis P., Carotenuto R.M., Pasanisi F., Cobellis L., Colacurci N. (2022). The Oncological Implication of Sentinel Lymph Node in Early Cervical Cancer: A Meta-Analysis of Oncological Outcomes and Type of Recurrences. Medicina.

[96] Bizzarri N., Querleu D., Ramirez P.T., Dostálek L., van Lonkhuijzen L.R.W., Giannarelli D., Lopez A., Salehi S., Ayhan A., Kim S.H. (2024). Survival associated with the use of sentinel lymph node in addition to lymphadenectomy in early-stage cervical cancer treated with surgery alone: A sub-analysis of the Surveillance in Cervical CANcer (SCCAN) collaborative study. Eur. J. Cancer.

[97] Favre G., Guani B., Balaya V., Magaud L., Lecuru F., Mathevet P. (2021). Sentinel Lymph-Node Biopsy in Early-Stage Cervical Cancer: The 4-Year Follow-Up Results of the Senticol 2 Trial. Front. Oncol..

[98] Brar H., Hogen L., Covens A. (2017). Cost-effectiveness of sentinel node biopsy and pathological ultrastaging in patients with early-stage cervical cancer. Cancer.

[99] Suidan R.S., Sun C.C.L., Cantor S.B., Frumovitz M., Giordano S.H., Meyer L.A. (2019). A cost-utility analysis of sentinel lymph node mapping versus complete lymphadenectomy in the management of early-stage cervical carcinoma. Gynecol. Oncol..

[100] Vicus D., Covens A. (2010). Role of sentinel lymph node biopsy in cervical cancer: Pro. Int. J. Gynecol. Cancer.

[101] Eiriksson L.R., Covens A. (2012). Sentinel lymph node mapping in cervical cancer: The future?. BJOG.

[102] Matsuura Y., Kawagoe T., Toki N., Tanaka M., Kashimura M. (2006). Long-standing complications after treatment for cancer of the uterine cervix--clinical significance of medical examination at 5 years after treatment. Int. J. Gynecol. Cancer..

[103] Biglia N., Librino A., Ottino M.C., Panuccio E., Daniele A., Chahin A. (2015). Lower limb lymphedema and neurological complications after lymphadenectomy for gynecological cancer. Int. J. Gynecol. Cancer.

[104] Hareyama H., Hada K., Goto K., Watanabe S., Hakoyama M., Oku K., Hayakashi Y., Hirayama E., Okuyama K. (2015). Prevalence, classification, and risk factors for postoperative lower extremity lymphedema in women with gynecologic malignancies: A retrospective study. Int. J. Gynecol. Cancer.

[105] Nakamura K., Kitahara Y., Satoh T., Takei Y., Takano M., Nagao S., Sekiguchi I., Suzuki M. (2016). Analysis of the effect of adjuvant radiotherapy on outcomes and complications after radical hysterectomy in FIGO stage IB1 cervical cancer patients with intermediate risk factors (GOTIC study). World J. Surg. Oncol..

[106] Bona A.F., Ferreira K.R., Carvalho R.B.M., Thuler L.C.S., Bergmann A. (2020). Incidence, prevalence, and factors associated with lymphedema after treatment for cervical cancer: A systematic review. Int. J. Gynecol. Cancer.

[107] Pieterse Q.D., Kenter G.G., Maas C.P., de Kroon C.D., Creutzberg C.L., Trimbos J.B.M.Z., Kuile M.M.T. (2013). Self-reported sexual, bowel and bladder function in cervical cancer patients following different treatment modalities: Longitudinal prospective cohort study. Int. J. Gynecol. Cancer.

[108] Sponholtz S.E., Ezendam N.P.M., de Rooij B.H., Parner E., Mogensen O., Hildebrandt M.G., Schledermann D., Markauskas A., Frøding L.P., Fuglsang K. (2022). SENTIREC—The sentinel node mapping in women with cervical cancer study—Patient-reported early lymphedema and its impact on quality of life. Gynecol. Oncol..

[109] Niikura H., Okamoto S., Otsuki T., Yoshinaga K., Utsunomiya H., Nagase S., Takano T., Ito K., Watanabe M., Yaegashi N. (2012). Prospective study of sentinel lymph node biopsy without further pelvic lymphadenectomy in patients with sentinel lymph node-negative cervical cancer. Int. J. Gynecol. Cancer.

[110] Lennox G.K., Covens A. (2017). Can sentinel lymph node biopsy replace pelvic lymphadenectomy for early cervical cancer?. Gynecol. Oncol..

[111] Gianoni M., Mathevet P., Uzan C., Bats A.S., Magaud L., Boutitie F., Lécuru F. (2020). Does the Sentinel Lymph Node Sampling Alone Improve Quality of Life in Early Cervical Cancer Management?. Front. Surg..

[112] Cibula D., Borčinová M., Marnitz S., Jarkovský J., Klát J., Pilka R., Torné A., Zapardiel I., Petiz A., Lay L. (2021). Lower-Limb Lymphedema after Sentinel Lymph Node Biopsy in Cervical Cancer Patients. Cancers.

[113] Balaya V., Bresset A., Guani B., Magaud L., Montero Macias R., Delomenie M., Bonsang-Kitzis H., Ngô C., Bats A.S., Mathevet P. (2020). Risk factors for failure of bilateral sentinel lymph node mapping in early-stage cervical cancer. Gynecol. Oncol..

[114] Kiss S.L., Stanca M., Căpîlna D.M., Căpîlna T.E., Pop-Suciu M., Kiss B.I., Kiss S.L., Căpîlna M.E. (2025). Sentinel Lymph Node Detection in Cervical Cancer: Challenges in Resource-Limited Settings with High Prevalence of Large Tumours. J. Clin. Med..

[115] Tu H., Wan T., Zhang X., Gu H., Feng Y., Huang H., Liu J. (2020). Potential risks in sentinel lymph node biopsy for cervical cancer: A single-institution pilot study. World J. Surg. Oncol..

[116] Kato H., Todo Y., Minobe S., Suzuki Y., Nakatani M., Ohba Y., Yamashiro K., Okamoto K. (2011). Previous conization on patient eligibility of sentinel lymph node detection for early invasive cervical cancer. Int. J. Gynecol. Cancer.

[117] Zhang M., Zhang Q., Wang X., Peng X., Chen J., Yang H. (2025). Prediction of clinical stages of cervical cancer via machine learning integrated with clinical features and ultrasound-based radiomics. Sci. Rep..

[118] Qin F., Sun X., Tian M., Jin S., Yu J., Song J., Wen F., Xu H., Yu T., Dong Y. (2024). Prediction of lymph node metastasis in operable cervical cancer using clinical parameters and deep learning with MRI data: A multicentre study. Insights Imaging.

[119] Luo S., Guo Y., Ye Y., Mu Q., Huang W., Tang G. (2025). Prediction of cervical cancer lymph node metastasis based on multisequence magnetic resonance imaging radiomics and deep learning features: A dual-center study. Sci. Rep..

[120] Baeten I.G.T., Hoogendam J.P., Stathonikos N., Gerestein C.G., Jonges G.N., van Diest P.J., Zweemer R.P. (2024). Artificial Intelligence-Based Sentinel Lymph Node Metastasis Detection in Cervical Cancer. Cancers.

